# Understanding the Stability of Salt-Inclusion Phases for Nuclear Waste-forms through Volume-based Thermodynamics

**DOI:** 10.1038/s41598-018-32903-3

**Published:** 2018-10-17

**Authors:** Emily E. Moore, Vancho Kocevski, Christian A. Juillerat, Gregory Morrison, Mingyang Zhao, Kyle S. Brinkman, Hans-Conrad zur Loye, Theodore M. Besmann

**Affiliations:** 1 Nuclear Engineering Program, Department of Mechanical Engineering, University of South Carolina, Columbia, SC 29208 USA; 2 Department of Chemistry, University of South Carolina, Columbia, SC 29208 USA; 3 Department of Materials Science and Engineering, Clemson University, Clemson, SC 29634 USA

## Abstract

Formation enthalpies and Gibbs energies of actinide and rare-earth containing SIMs with silicate and germanate frameworks are reported. Volume-based thermodynamics (VBT) techniques complemented by density functional theory (DFT) were adapted and applied to these complex structures. VBT and DFT results were in closest agreement for the smaller framework silicate structure, whereas DFT in general predicts less negative enthalpies across all SIMs, regardless of framework type. Both methods predict the rare-earth silicates to be the most stable of the comparable structures calculated, with VBT results being in good agreement with the limited experimental values available from drop solution calorimetry.

## Introduction

Nuclear waste sequestration, including legacy materials from weapons programs as well as spent fuel from research reactors and potential commercial fuel recycling remains an important contemporary issue. While many reprocessing techniques exist, and repository solutions have been proposed, there is still a large research focus on how to more effectively and efficiently immobilize certain problematic radionuclides, especially those which are easily volatilized or for which waste glass loading is limited. A novel approach to simultaneously capturing multiple nuclear waste products includes the use of hierarchical architectures of porous materials. The working definition of a hierarchical material is that of a structural motif contained within a larger structure or framework. A class of materials that exhibit this structural characteristic include salt inclusion materials (SIMs).

Salt-inclusion materials exhibit a hierarchical structure that consists of a covalent mixed-oxide framework which contains a void filled with simple ionic salts. While traditional SIMs are characterized by transition metal oxides interconnected with oxyanion units of groups 14 and 15 elements such as Si, Ge, As, P^1–5^ more recently, uranyl^6–9^ and lanthanide^10^ salt-inclusion phases are being explored for nuclear waste applications due to their porous or “stuffed” nature. The framework allows for structural variability forming uranyl-based silicate, germanate, vanadate, phosphate or borate networks with various 3-D void sizes, which are filled with ionic salts that preferentially contain radionuclides. The general description of uranyl SIMs is the structural formula [A_m_B_n_X][(UO_2_)_p_(M_q_O_r_)_t_], where [(UO_2_)_p_(M_q_O_r_)_t_] is the framework consisting of uranyl cations, UO_2_^2+^, and M_q_O_r_ units (M = network forming ion such as Si or Ge), B_n_X is the salt-inclusion, and A are non-salt-inclusion cations. To widen the class of materials, ion exchange in SIMs can be performed to include targeted isotopic compositions.

Preparation of the framework materials take size and charge variations into account during synthesis; however, little is known about their thermodynamic stability, including formation enthalpies or Gibbs energies. For known phases, calorimetric methods can provide a direct measure of the formation energy of the materials, however to date there is no published literature on the thermodynamic properties of SIMs. Predictive thermodynamics is an attractive technique as it can provide insight into the thermodynamic stability of novel new structures such as SIMs, as well as guide the synthesis of newly formulated materials. Volume-based thermodynamics (VBT) is a tool developed by Glasser *et al*.^11–13^ which serves to estimate thermodynamic parameters of a class of newly synthesized or even hypothetical materials when experimental thermochemical data are lacking and other theoretical modeling and simulation techniques are uncertain and can be computationally prohibitive. In this work we aim to provide a library of Gibbs energy values for a set of systems that encompass a multitude of different structural frameworks and potential salt inclusions to effectively inform the sequestration of radionuclides for waste management. To our knowledge this is the first attempt to apply VBT to complex hierarchical structures such as salt-inclusion materials.

## Methods

### Volume based thermodynamics (VBT)

**Figure 1:**
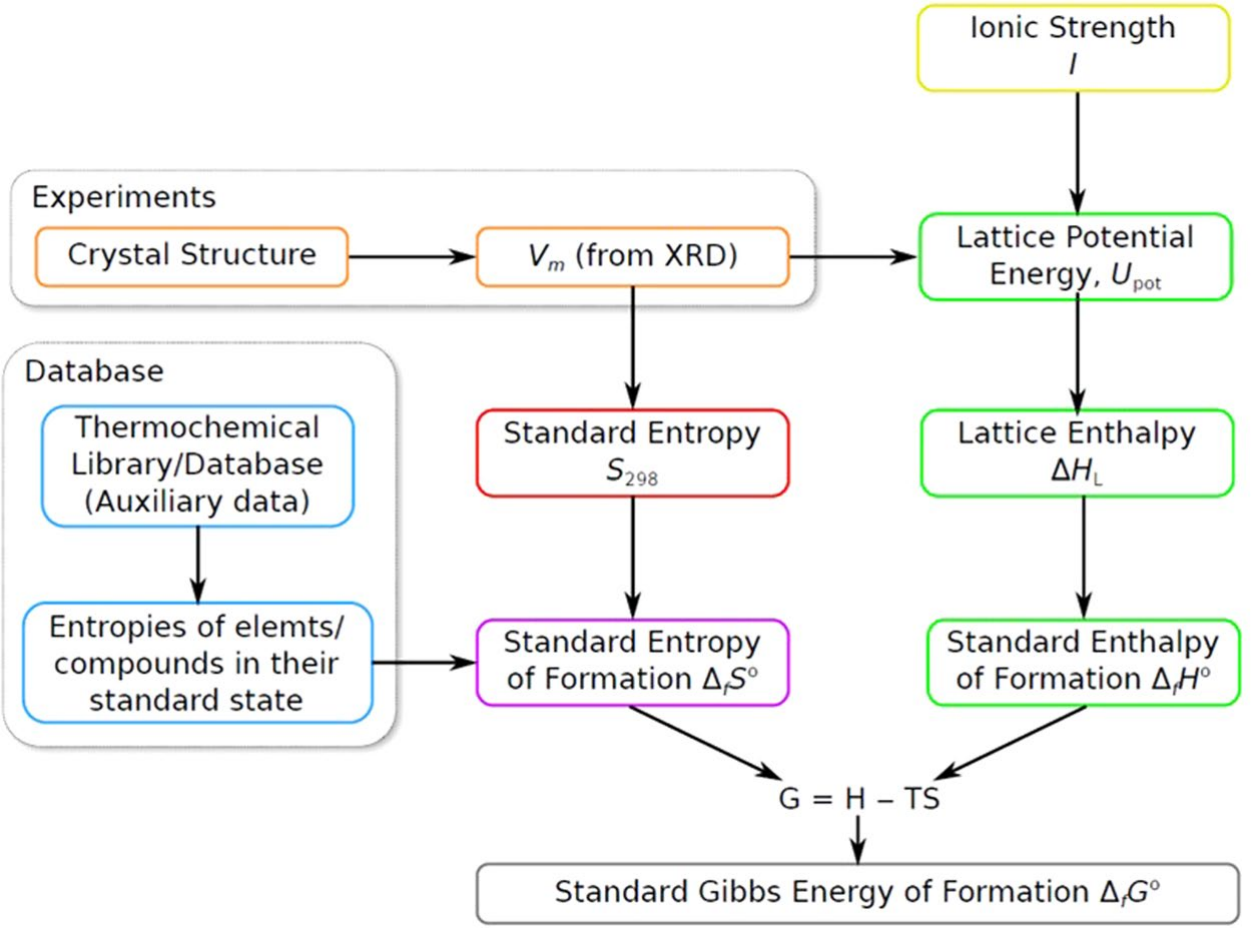
Schematic for calculating thermodynamic values from VBT methods.

The VBT method incorporates empirical relations to generate estimated quantities of the standard entropy (*S*°_298.15_), enthalpy of formation (Δ_*f*_*H*°_298.15_) and Gibbs energy of formation (Δ_*f*_*G*°_298.15_. The method uses crystallographic information from X-ray diffraction or density measurements if the formula mass is known, to obtain the volume per formula unit (V_m_). In this work the formula unit volume is calculated by dividing the volume of the unit cell V_cell_ (from a crystallographic information file; CIF) by the number of formula units Z in the unit cell so that V_m_ = V_cell_/Z. This quantity is then used in conjunction with derived thermodynamic cycles to calculate the formation energetics, as presented in the schematic of Fig. 1.

The standard entropy is calculated with Eq. 1, where the fitted constants **k** (J/K/mol/nm^3^) and **c** (J/mol/K) are applied with the formula unit volume, with the constants varying as to whether the system is organic (liquid or solid) or ionic (hydrous or anhydrous). In this case we take the constants as fitted for anhydrous ionic salts^12^.

1 $${{S}}_{298.15}^{^\circ }={k}{{V}}_{m}+{c}$$

A lattice potential energy is required which is calculated from Eq. 2 and is indicative of the ability of an ionic solid to form from components in the gaseous state, where the ionic strength factor ***I*** $({I}=1/2\,\sum _{{i}}{{n}}_{{i}}{{z}}_{{i}}^{2})$ is calculated from the constituents of the salt and the salt-inclusion framework and their respective charges, with ***n***_***i***_ being the number of ion types, ***z***_***i***_ their respective charge; and ***A*** the standard electrostatic Madelung constant (121.39 nm kJ/mol)^11,12^.

2 $${{U}}_{{\rm{pot}}}={A}\,{I}\,{(2{I}/{{V}}_{m})}^{1/3}$$

The lattice energy is then converted into a useable enthalpic value by a multiplicative RT term that includes information on the ion types (**s**_**i**_) and a constant (**c**_**i**_) related to whether the ion types are monoatomic, polyatomic (linear or non-linear) as shown in Eq. 3, with ***R*** being the ideal gas constant and ***T*** the temperature in Kelvin.

3 $${\rm{\Delta }}{{H}}_{L}={{U}}_{{\rm{pot}}}+\sum _{{i}=1}^{{n}}{{s}}_{{i}}(\frac{{{c}}_{{i}}}{2}-2){RT}\,$$

The Born-Haber-Fajans cycle, which applies Hess’ law is then used to calculate the standard enthalpy of formation in which the constituents of the salt-inclusion material are broken down into their gaseous ionic counterparts, where the salt inclusion components are broken down into their elemental state, and the framework consists of constituents in various oxide forms. Information regarding the gaseous components from the solid phase are obtained from auxiliary information in Table 1 and include enthalpies of sublimation or dissociation, combined with ionization potentials (IP) or electron affinities (EA) for cationic and anionic species respectively, which are found in the literature. The summation of these energies in the gas state along with the lattice enthalpy (Eq. 4) results in a value for the standard enthalpy of formation. The latter value then allows for the calculation of the Gibbs energy of formation by applying auxiliary information for the standard entropy to Eq. 1.

4 $${{\rm{\Delta }}}_{{\boldsymbol{f}}}{H}{^\circ }_{298.15}={\rm{\Delta }}{{H}}_{{\rm{sub}}}+{\rm{IP}}+{\rm{\Delta }}{{H}}_{{\rm{dis}}}+{\rm{EA}}+{\rm{\Delta }}{{H}}_{L}$$

**Table 1:** Collection of auxiliary data for use in Born-Haber-Fajans cycle.

Species	Δ_*f*_*H*_*gas*_/Δ*H*_*sub*_	IP	IP (2nd)	IP (3rd)	Δ*H*_*dis*_	EA	*S*°_298.15_
	[kJ/mol]	[kJ/mol]	[kJ/mol]	[kJ/mol]	[kJ/mol]	[kJ/mol]	[J/mol/K]
UO_2_ (s)	−462.1^27^	591.3^28,29^	1380^29,30^	—	—	—	77.03^27^
Gd	406.9^31^	593.4^28^	1166.5^28^	1990.5^28^	—	—	68.1^31^
Eu	178.2^31^	547.1^28^	1084.6^28^	2404.4^28^	—	—	77.8^31^
SiO_2_ (s)	−305.4^32^	—	—	—	—	−195.9^33^	41.5^32^
Si_2_O_5_^2−^ (g)	−1833.9^DFT^	—	—	—	—	—	—
GeO_2_ (s)	−106.2^34^	—	—	—	—	−241.2^35^	39.7^36,37^
GeO (g)	−37.7^34^	—	—	—	—	−13.8^38^	—
Ge_2_O_5_^2−^ (g)	−1644.7^DFT^	—	—	—	—	—	—
O_2_ (g)	0	—	—	—	493.6^37^	−42.5^32^	205.2^32^
O (g)	249.2^32^	—	—	—	—	−141.0^32^	161.1^32^
Na (s)	107.3^32^	495.8^32^	—	—	—	—	51.46^32^
K (s)	89.0^32^	418.8^32^	—	—	—	—	65.67^32^
Rb (s)	80.9^32^	403.0^32^	—	—	—	—	76.78^32^
Cs (s)	76.5^32^	375.7^32^	—	—	—	—	85.15^32^
Ag (s)	284.8^39^	731.0^40^	—	—	157.7^39^	—	42.48^39^
F_2_ (g)	0	—	—	—	154.6^32^	−328.0^32^	202.8^32^
Cl_2_ (g)	0	—	—	—	239.2^32^	−349.0^32^	223.1^32^
Br_2_ (g)	0	—	—	—	190.2^32^	−324.7^32^	152.2^32^

A mixing entropy accounts for the combining of the different components of the salt, where contributions of partially occupied and mixed salts are naturally greater than those with a single cation type. The relation is seen in Eq. 5, where ***n*** is the total number of moles and ***x***_***i***_ is the mole fraction of each constituent.

5 $${{S}}_{{mix}}=-\,{nR}\sum _{{i}}{{x}}_{{i}}\,{ln}({{x}}_{{i}})$$

The VBT approach was applied to three different classes of salt-inclusion frameworks: Uranyl silicates (9 compounds) uranyl germanates (13 compounds) and rare-earth silicates (2 compounds). The compositions were obtained from the literature or synthesized by the methods described in^9,14^, and are listed in Table 2 along with V_m_ values derived from available crystallographic information.

**Table 2:** List of SIMs treated using VBT, along with the crystallographic data to calculate the formula unit volume (V_m_).

Salt inclusion structure	V_cell_ (Å^3^)	Z	V_m_ (Å^3^)
[Cs_3_F][(UO_2_)(Si_4_O_10_)]^9^	1542.68	4	385.7
[Cs_9_Cs_6_Cl][(UO_2_)_7_(Si_6_O_17_)_2_(Si_4_O_12_)]^9^	1890.08	1	1890.1
[NaK_6_F][(UO_2_)_3_(Si_2_O_7_)_2_]^8^	1139.71	2	569.9
[KK_6_Cl][(UO_2_)_3_(Si_2_O_7_)_2_]^8^	1184.82	2	592.4
[NaRb_6_F][(UO_2_)_3_(Si_2_O_7_)_2_]^7^	1187.73	2	593.9
[K_3_Cs_4_F][(UO_2_)_3_(Si_2_O_7_)_2_]^7^	2451.13	4	612.8
[Cs_2_Cs_5_F][(UO_2_)_3_(Si_2_O_7_)_2_]^9^	1382.41	2	691.2
[Cs_2_Cs_5_F][(UO_2_)_2_(Si_6_O_17_)]^9^	1436.05	2	718.0
[Na_9_F_2_][(UO_2_)(UO_2_)_2_(Si_2_O_7_)_2_]^5^	516.53	1	516.5
[Cs_2_Cs_5_F][(UO_2_)_3_(Ge_2_O_7_)_2_]^14^	1451.65	2	725.83
[Cs_6_ Ag_2_Cl_2_][(UO_2_)_3_(Ge_2_O_7_)_2_]^14^	1450.41	2	725.21
[Cs_6_ Ag_0.3_Na_1.7_Cl_2_][(UO_2_)_3_(Ge_2_O_7_)_2_]^14^	1444.51	2	722.26
[Cs_6_ Ag_0.4_Na_1.6_Cl_2_][(UO_2_)_3_(Ge_2_O_7_)_2_]^14^	1445.17	2	722.59
[Cs_6_K_2_Cl_2_][(UO_2_)_3_(Ge_2_O_7_)_2_]^14^	1460.71	2	730.36
[Cs_6_K_1.9_Ag_0.1_Cl_2_][(UO_2_)_3_(Ge_2_O_7_)_2_]^14^	1476.60	2	738.30
[KK_6_Cl][(UO2)3(Ge_2_O_7_)_2_]^14^	1257.44	2	628.72
[KK_6_Br_0.6_F_0.4_][(UO_2_)_3_(Ge_2_O_7_)_2_]^14^	1263.60	2	631.80
[Na_0.9_Rb_6.1_F][(UO_2_)_3_(Ge_2_O_7_)_2_]^14^	1261.39	2	630.70
[K_0.6_Na_0.4_K_5_CsCl_0.5_F_0.5_][(UO_2_)_3_(Ge_2_O_7_)_2_]^14^	1258.66	2	629.33
[K_0.8_Na_0.2_K_4.8_Cs_1.2_Cl_0.5_F_0.5_][(UO_2_)_3_(Ge_2_O_7_)_2_]^14^	1264.30	2	632.15
[KK_1.8_Cs_4.2_F][(UO_2_)_3_(Ge_2_O_7_)_2_]^14^	2612.41	4	653.10
[Cs_6_Cs_0_._71_Cl_0.71_][(UO_2_)_3_(Ge_2_O_7_)O_3_]^14^	1294.40	2	647.20
[K_2_K_7_F_2_] [Eu_3_Si_12_O_32_]^10^	888.39	1	888.39
[K_2_K_7_F_2_][Gd_3_Si_12_O_32_]^10^	888.87	1	888.87

### Density functional theory (DFT)

The DFT calculations were performed using the code VASP^15,16^, with the Perdew-Burke-Ernzerhof (PBE) generalized-gradient approximation^17^, employing the projector augmented plane wave (PAW) method^18,19^. For calculating the enthalpies of formation of Si_2_O_5_^2−^ and Ge_2_O_5_^2−^ we considered the systems to be composed of a 2D sheet formed by two SiO_4_ and GeO_4_ tetrahedra, with three corner sharing O atoms and a −2*e* charge. Considering that the U atoms are surrounded by O atoms, we chose a value of *U*_eff_ = 4.0 eV, which is a *U*_eff_ value that is close to that obtained from experimental studies for UO_2_^20,21^ and has been proven to well-reproduce the structural parameters and band gaps of for UO_3_ polymorphs^22–24^. The calculations were performed using 12 × 12 × 1 *k*-point mesh, 520 eV cutoff energy for the planewave basis set, and 10^−8^ eV and 0.001 Å/eV energy and forces convergence criteria, respectively, allowing the systems to fully relax (volume, cell shape and ionic positions). For the SIMs the calculations utilized a 500 eV planewave energy cutoff, 10^−6^ energy convergence criteria, *k*-point mesh with 3000 KPPRA (k-point density per reciprocal atom), and fully relaxed systems.

### Thermochemical cycles

**Figure 2:**
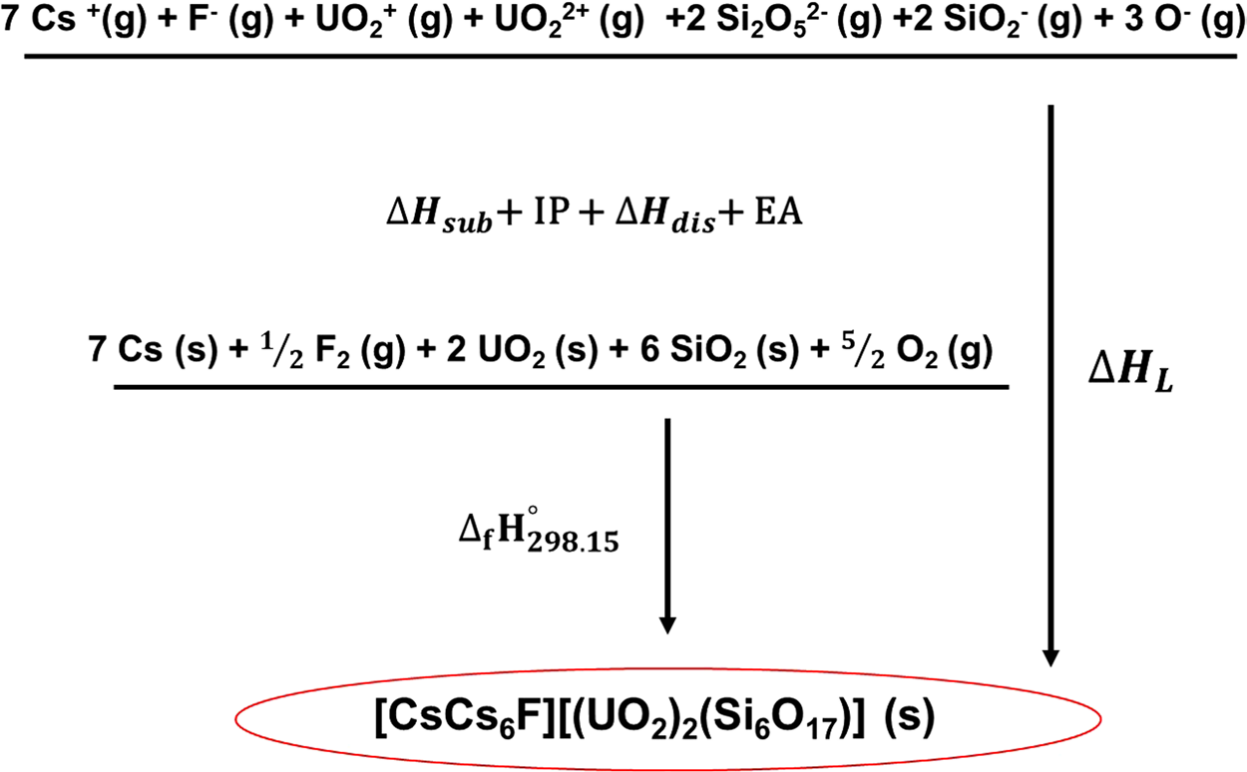
Thermochemical cycle for a uranyl silicate salt inclusion.

Each of the SIMs frameworks are broken down into individual constituents based on the available auxiliary information, where silicate and germanate oxide constituents are initially limited to SiO_2_/GeO_2_ and SiO/GeO components with a single negative charge. To obtain a better representation of the silicate SiO_4_, and germanate GeO_4_ tetrahedra, which often arrange in Si_4_O_10_ and Ge_4_O_10_ columns, the components Si_2_O_5_^2−^(g) and Ge_2_O_5_^2−^ are needed and thus density functional theory (DFT) calculations were performed to calculate the formation enthalpy of these constituents for which no information is available. The anion frameworks are charge-balanced by varying the oxidation state of uranium in the uranyl cations so that the overall salt-framework is neutral. An example of a balanced Born-Haber-Fajans cycle used to calculate the Δ_*f*_*H*°_298.15_ is depicted in Fig. 2. The remaining constituents that make up the various silicate, germanate and rare-earth framework cycles are reported in Table 3, where the single-ion values that make up the salt-inclusions are directly taken from the auxiliary data table.

**Table 3:** Thermochemical cycles for SIMs framework components.

Framework Structure	Charge	Thermocycle components
[(UO_2_)(Si_4_O_10_)]	2−	UO_2_^2+^ (g) + 2 Si_2_O_5_^2−^ (g)
[(UO_2_)_7_(Si_6_O_17_)_2_(Si_4_O_12_)]	14−	4 UO_2_^+^ (g) + 3 UO_2_^2+^ (g) + 6 Si_2_O_5_^2−^ (g) + 4 SiO_2_^−^ (g) + 8 O^−^ (g)
[(UO_2_)_3_(Si_2_O_7_)_2_]	6−	3 UO_2_^+^ + Si_2_O_5_^2−^ (g) + 2 SiO_2_^−^ (g) + 5 O^−^ (g)
[(UO_2_)_2_(Si_6_O_17_)]	6−	UO_2_^+^ (g) + UO_2_^2+^ (g) + 2 Si_2_O_5_^2−^ (g) + 2 SiO_2_^−^ (g) + 3 O^−^ (g)
[(UO_2_)(UO_2_)_2_(Si_2_O_7_)_2_]	7−	3 UO_2_^+^ (g) + 4 SiO_2_^−^ (g) + 6 O^−^ (g)
[(UO_2_)_3_(Ge_2_O_7_)_2_]	6−	UO_2_^2+^ (g) + 2 UO_2_^+^ (g) + 4 GeO_2_^−^ (g) + 6 O^−^ (g)
[(UO_2_)_3_ O_3_(Ge_2_O_7_)]	6−	3 UO_2_^+^ (g) + GeO_2_^−^(g) + GeO^−^ (g) + 7 O^−^ (g)
[Ln_3_Si_12_O_32_] (Ln = Eu or Gd)	7−	2 Ln^2+^ (g) + Ln^3+^ (g) + 6 Si_2_O_5_^2−^ (g) + 2 O^−^ (g)

## Results

**Figure 3:**
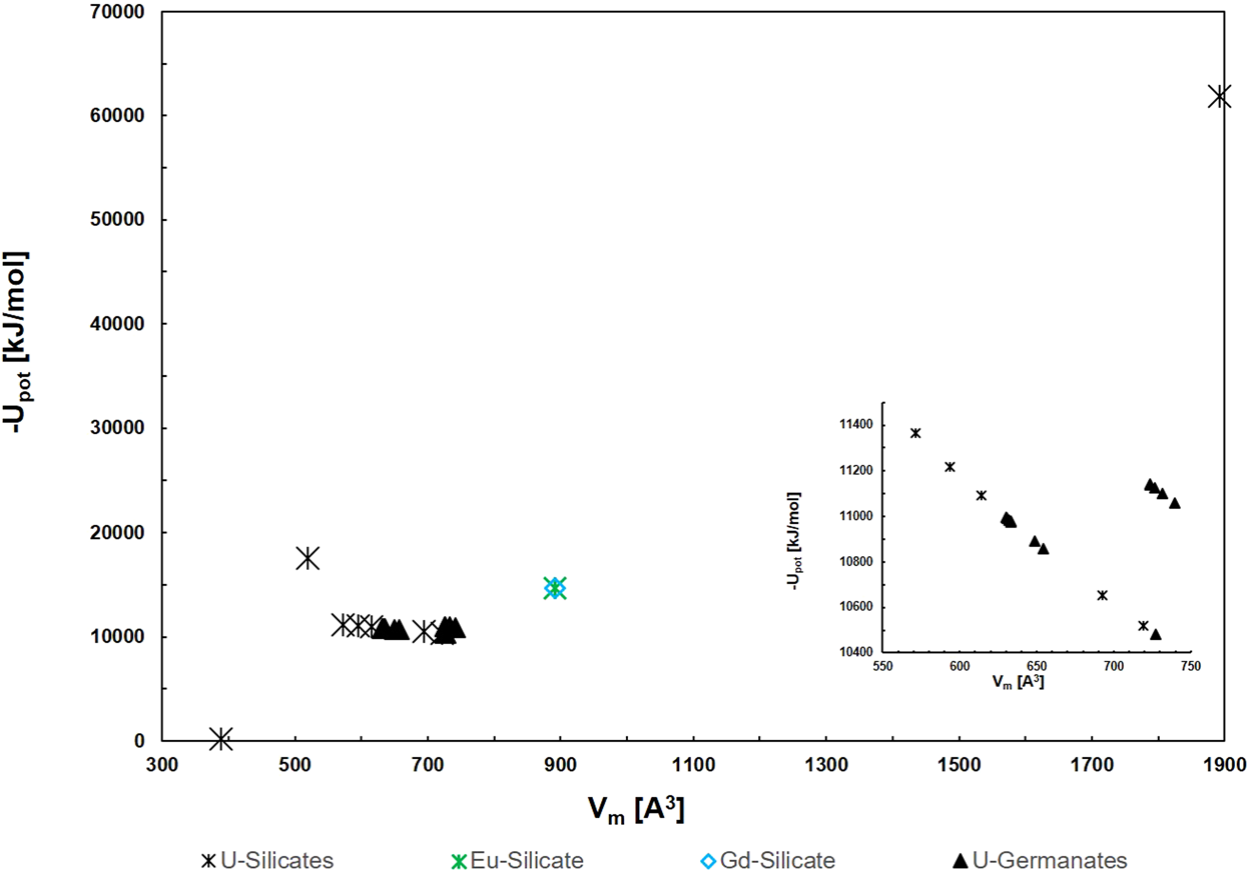
Lattice potential energy (U_pot_) as a function of V_m_ for SIMs, the inset shows the Ge and Si frameworks with V_m_ between 550–750 Å^3^.

**Figure 4:**
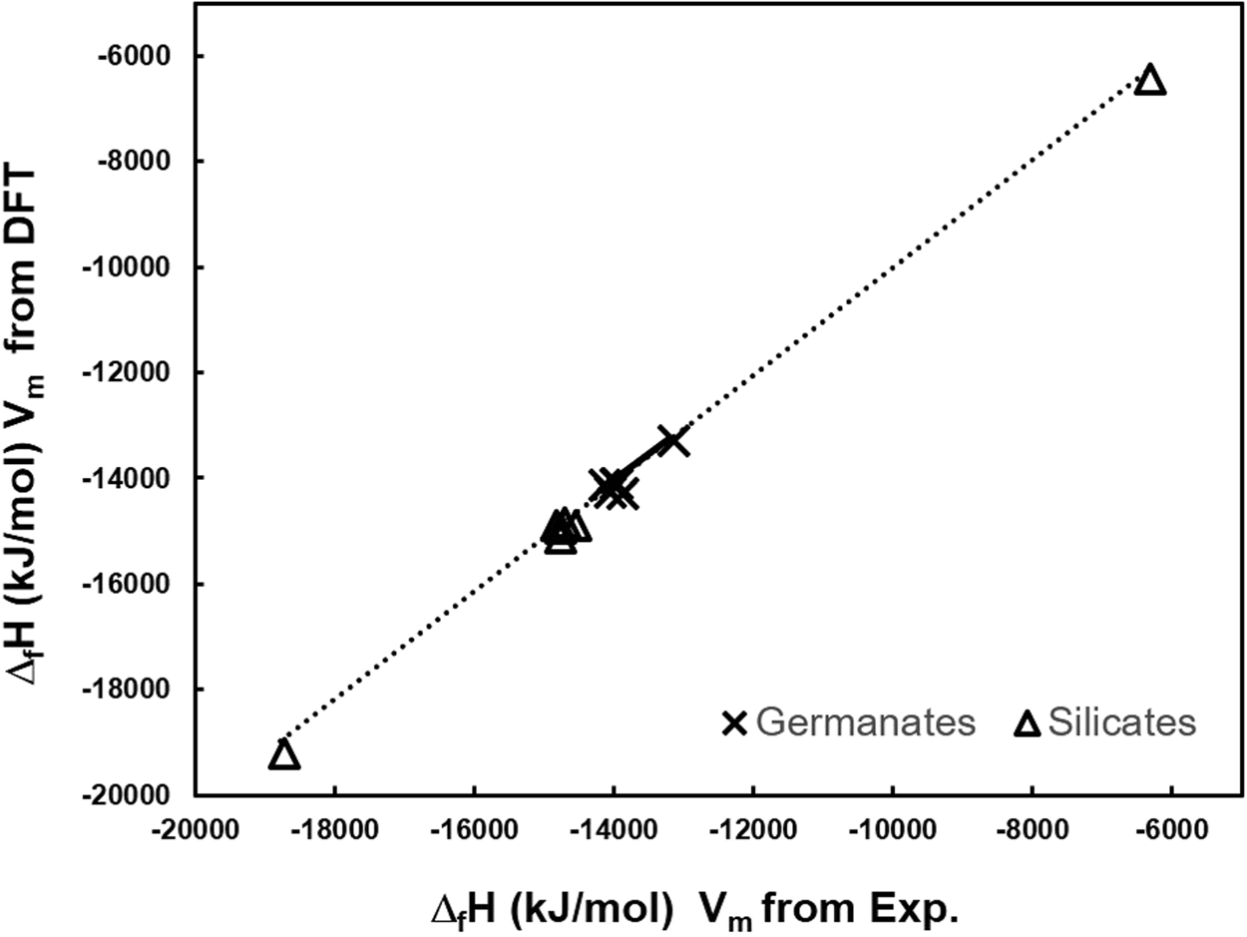
VBT computed formation enthalpies using experimental and DFT calculated V_m_.

**Figure 5:**
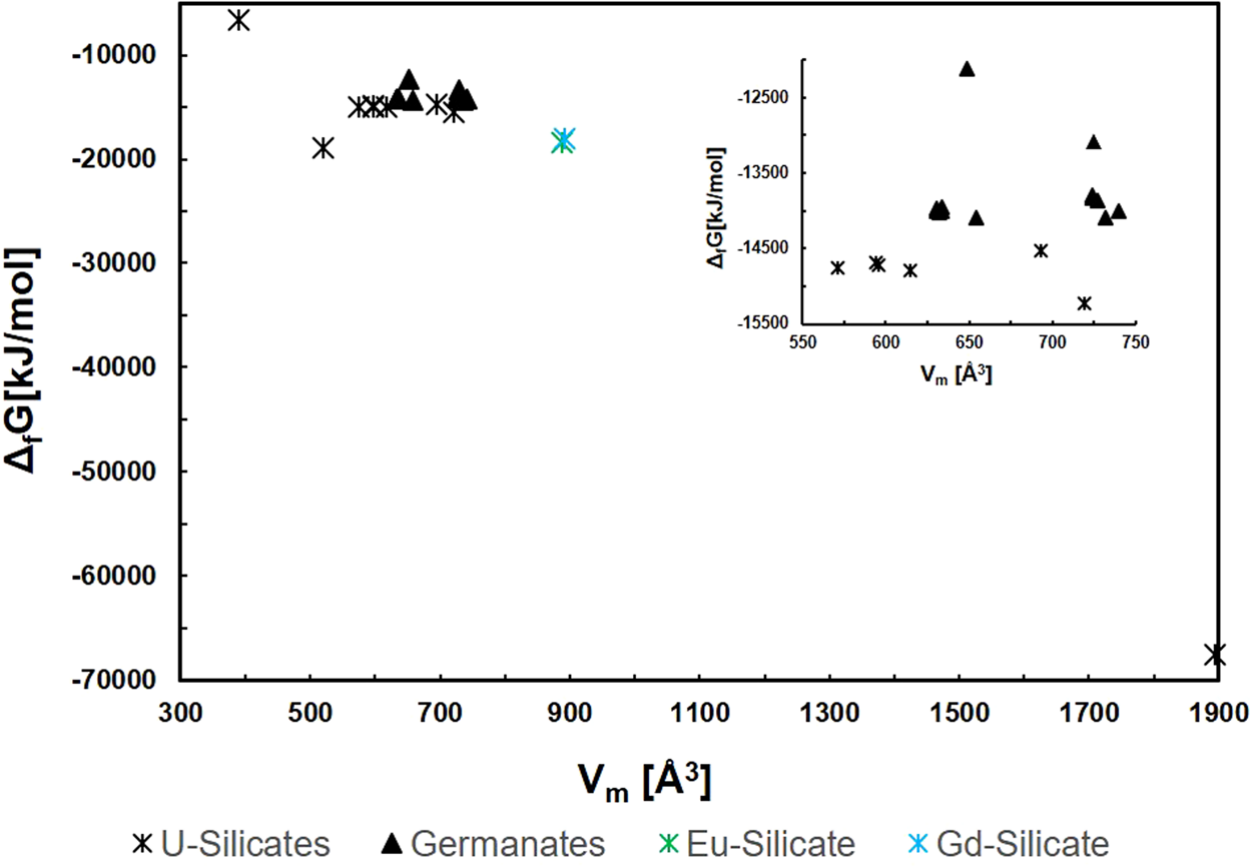
Gibbs energy of formation as a function of V_m_ for silicate and germanate SIMs, the inset shows the Ge and Si frameworks with V_m_ between 550–750 Å^3^.

The lattice potentials calculated using Eq. 2 are plotted as a function of the formula unit volume for the available SIMs in Fig. 3. The uranyl silicate materials include more versatile framework structures, where different charged frameworks and salts are considered. Both the lanthanoid (Ln) silicates and uranyl germanates (except for one structure) have the exact same framework composition. The increased variance of the salt inclusions, including their charge and composition, allows for a range of differently charged uranyl-silicate frameworks, which dictates the lattice stability, which is largely dependent on the ionic strength factor. Conversely, the germanate SIMs have identical frameworks for twelve of the thirteen structures. For both silicates and germanates with self-same frameworks, the lattice potential decreases with increasing V_m_, as it is inversely proportional to its cube root of the value (see Eq. 2) and the ionic strength factor is less influential due to the similarity of the salt-inclusions. The Δ_*f*_*H*°_298.15_ are calculated using the auxiliary information in Table 1 and are compared with experiment and values calculated by DFT in Table 4. Only salt inclusions which did not have partial occupancies were computed by DFT as the significantly larger unit cell required for considering partial occupancies made the calculations prohibitively computationally intensive. The Δ_*f*_*H*°_298.15_ value was also calculated with VBT using volumes derived from DFT relaxed structures, the energies are compared in Fig. 4. The VBT Δ_*f*_*H*°_298.15_ values plus the standard entropy calculated from Eq. 1 provide the Gibbs energy of formation, both of which are listed in Table 4 and the latter depicted in Fig. 5. The energies include the mixing entropy of the salt-components as noted above and as was demonstrated in Juillerat *et al*.^25^ for alkali metals.

**Table 4:** Enthalpies of formation (kJ/mol), Gibbs energies of formation (kJ/mol) and standard entropies (J/mol/K) of SIMs from VBT compared with DFT and Experiment.

Salt inclusion structure	Δ_*f*_*H*°_298.15_(VBT)	Δ_*f*_*H*°_0*K*_	Δ_*f*_*H*°_298.15_(Exp)	*S*°_298.15_	Δ_*f*_*G*°_298.15_
[Cs_3_F][(UO_2_)(Si_4_O_10_)]	−6361	−5719		539.5	−6344
[Cs_9_Cs_6_Cl][(UO_2_)_7_(Si_6_O_17_)_2_(Si_4_O_12_)]	−67501			2585.5	−67346
[NaK_6_F][(UO_2_)_3_(Si_2_O_7_)_2_]	−14833	−9297		790.0	−14717
[KK_6_Cl][(UO_2_)_3_(Si_2_O_7_)_2_]	−14762	−9214		820.7	−14644
[NaRb_6_F][(UO_2_)_3_(Si_2_O_7_)_2_]	−14821	−9368		822.7	−14693
[K_3_Cs_4_F][(UO_2_)_3_(Si_2_O_7_)_2_]	−14879	−9254		848.4	−14757
[Cs_2_Cs_5_F][(UO_2_)_3_(Si_2_O_7_)_2_]	−14609			955.0	−14488
[Cs_2_Cs_5_F][(UO_2_)_2_(Si_6_O_17_)]	−15262	−9690		991.5	−15185
[Na_9_F_2_][(UO_2_)(UO_2_)_2_(Si_2_O_7_)_2_]	−18782	−9930		717.5	−18616
[Cs_2_Cs_5_F][(UO_2_)_3_(Ge_2_O_7_)_2_]*	−13931	−7909		1002.1	−13826
[Cs_6_ Ag_2_Cl_2_][(UO_2_)_3_(Ge_2_O_7_)_2_]*	−13202	−7760		1001.3	−13084
[Cs_6_ Ag_0.3_Na_1.7_Cl_2_][(UO_2_)_3_(Ge_2_O_7_)_2_]*	−13919			997.3	−13797
[Cs_6_ Ag_0.4_Na_1.6_Cl_2_][(UO_2_)_3_(Ge_2_O_7_)_2_]*	−13876			997.7	−13755
[Cs_6_K_2_Cl_2_][(UO_2_)_3_(Ge_2_O_7_)_2_]*	−14192	−8338		1008.3	−14063
[Cs_6_K_1.9_Ag_0.1_Cl_2_][(UO_2_)_3_(Ge_2_O_7_)_2_]*	−14101			1019.1	−13977
[KK_6_Cl][(UO2)_3_(Ge_2_O_7_)_2_]^¥^	−14035	−7870		870.1	−13931
[KK_6_Br_0.6_F_0.4_][(UO_2_)_3_(Ge_2_O_7_)_2_]^¥^	−14017	−7914		874.2	−13923
[Na_0.9_Rb_6.1_F][(UO_2_)_3_(Ge_2_O_7_)_2_]^¥^	−14105	−8012		872.7	−13992
[K_0.6_Na_0.4_K_5_CsCl_0.5_F_0.5_][(UO_2_)_3_(Ge_2_O_7_)_2_]^¥^	−14060			870.9	−13965
[K_0.8_Na_0.2_K_4.8_Cs_1.2_Cl_0.5_F_0.5_][(UO_2_)_3_(Ge_2_O_7_)_2_]^¥^	−14074			874.7	−13977
[KK_1.8_Cs_4.2_F][(UO_2_)_3_(Ge_2_O_7_)_2_]^¥^	−14151			903.2	−14049
[Cs_6_Cs_0.71_Cl_0.71_][(UO_2_)_3_(Ge_2_O_7_)O_3_]^§^	−12202			895.2	−12082
[K_2_K_7_F_2_] [Eu_3_Si_12_O_32_]	−18594	−16267		1223.2	−18436
[K_2_K_7_F_2_][Gd_3_Si_12_O_32_]	−17935	−15978	−17389 [16]	1223.9	−17725

*Monoclinic, ^¥^orthorhombic, ^§^hexagonal (distinctions are made for germanates of equal charged frameworks).

## Discussion

The results in Table 4 indicate relative good agreement between DFT and VBT values for the formation enthalpy of [Cs_3_F][(UO_2_)(Si_4_O_10_)], whereas the formation enthalpies for the other uranyl-silicates derived using VBT are much more negative (more thermodynamically stable) than those calculated from DFT. However, both methods predict the following trend in framework energetics:

$${[({{\rm{UO}}}_{2})({{\rm{Si}}}_{4}{{\rm{O}}}_{10})]}^{2-} < {[{({{\rm{UO}}}_{2})}_{3}{({{\rm{Si}}}_{2}{{\rm{O}}}_{7})}_{2}]}^{6-} < {[{({{\rm{UO}}}_{2})}_{2}({{\rm{Si}}}_{6}{{\rm{O}}}_{17})]}^{6-} < {[({{\rm{UO}}}_{2}){({{\rm{UO}}}_{2})}_{2}{({{\rm{Si}}}_{2}{{\rm{O}}}_{7})}_{2}]}^{7-}$$

This indicates that for the silicates, the charge on the framework (which contributes to a higher ionic strength factor) and the overall size of the system (such as the total number of atoms per formula unit), influences the thermodynamic stability. More negatively charged frameworks that allow for larger salt inclusions have a more negative enthalpy of formation. With equivalently charged frameworks, the silicon-rich system is found to be more stable than its uranium-rich counterpart, according to both DFT and VBT. The VBT values for [(UO_2_)_3_(Si_2_O_7_)_2_]^6−^ and [(UO_2_)_2_(Si_6_O_17_)]^6−^ framework types with identical Cs_2_Cs_5_F salt inclusion, imply that the silicon-rich composition is more thermodynamically stable (has a more negative formation enthalpy). While the increased negative value in formation enthalpy (+4.3%) might be attributed to the increase in ***V***_***m***_ (+3.8%) for the silicon-rich framework, it seems more likely that the choice of constituents for the utilized thermodynamic cycle are more influential. In the case of the silicon rich [(UO_2_)_2_(Si_6_O_17_)]^6−^ framework the cycle includes the use of ${{\rm{UO}}}_{2}^{2+}$, which has a greater impact on the formation energetics, since both the first and second ionization potentials are included. The silicon rich framework allows for a better representation of the structure by including both Si and U in their proper Si^4+^ and U^6+^ oxidation states respectively. This work attempts to use U^VI^ ions in the thermodynamic cycles whenever possible as it is a more realistic description of the system, since all frameworks but one contain this oxidation state of the uranyl cation. Nevertheless, given the limitations of the auxiliary information, ${{\rm{UO}}}_{2}^{2+}$ is not always represented as such in the VBT cycles. As indicated in Table 3, in order to properly charge-balance the system, only a singly charged uranyl cation (UO_2_^+^) is often used.

Equivalent frameworks in both composition and charge differ only in the salt-inclusion which dictates ***V***_***m***_, where the Cs_2_Cs_5_F salt-inclusion results in a much larger ***V***_***m***_ (15.4%) compared to the other four NaK_6_F, KK_6_Cl, Na_0.9_Rb_6.1_F, K_3_Cs_4_F salt compositions. The average framework ***V***_***m***_ was calculated as 508.0 ± 23.3 Å^3^, where the thermochemical radii of the alkali metals and halides are used to compute the ***V***_***m***_ of the salt-inclusions. The volume of the salt is then subtracted from the overall formula unit volume of the five identical framework materials, which are then averaged. The larger formula unit volume of the pure cesium containing (Cs_2_Cs_5_F) SIM leads to a formation enthalpy that is less negative than its four counterparts; a similar trend was found in^25^, where larger alkali inclusions (and therefore ***V***_***m***_ values) resulted in less negative formation enthalpies. For the remaining SIMs of the [(UO_2_)_3_(Si_2_O_7_)_2_]^6−^ family, both DFT and VBT predict that the chlorine containing KK_6_Cl salt is the least stable structure and the NaK_6_F salt-inclusion is the second most stable structure. DFT predicts the NaRb_6_F to have the most negative formation enthalpy, whereas VBT predicts the mixed K_3_Cs_4_F salt to be the most stable. A similar result was obtained for the mixed KK_1.8_Cs_4.2_F salt in the monoclinic germanate framework presented below.

The uranyl germanate framework, [(UO_2_)_3_(Ge_2_O_7_)_2_]^6−^, is analogous to the silicate framework, [(UO_2_)_3_(Si_2_O_7_)_2_]^6−^, and twelve different salt inclusions have been incorporated into this framework producing structures in either the orthorhombic or monoclinic setting. A lone hexagonal structure with a different framework, [(UO_2_)_3_O_3_(Ge_2_O_7_)]^6−^ has also been synthesized (the experimental results of all uranyl germanate SIMs are detailed in^14^). The enthalpies of formation of the DFT and VBT values are listed in Table 4 and overall are less negative than those for the silicates with a similar framework composition. DFT values predict the average formation enthalpies of the [(UO_2_)_3_(Si_2_O_7_)_2_]^6−^ silicates (−9365 kJ/mol) to be more negative by 16.1% than the [(UO_2_)_3_(Ge_2_O_7_)_2_]^6−^ germanates (−7967 kJ/mol), whereas VBT predicts a difference of 5.6% between the silicates (−14781 kJ/mol) and germanates (−13972 kJ/mol). Yet the effects of the choice of constituents for the thermochemical cycles, i.e., using GeO^−^/GeO_2_^−^ and SiO^−^/SiO_2_^−^ reveals that large discrepancies can arise. This highlights the importance and limitations of the auxiliary information when calculating the thermodynamic cycles, especially the need to charge balance the framework components.

VBT predicts the orthorhombic structures in general to be slightly more stable than monoclinic structures. This could in part be due to the symmetry of the structures (i.e., orthorhombic crystal systems have higher symmetry than monoclinic) or the difference in the salt-inclusions. All of the systems with monoclinic symmetry consist of dihalide salts (except for the Cs_2_Cs_5_F) and are cesium rich, whereas the orthorhombic structures generally incorporate less cesium and exclusively include only single halide salts. For the monoclinic structures calculated by DFT, the trends in relative stability are in agreement with the results from VBT, such that the silver containing structure is the least stable, followed by the pure cesium compound. As with the silicates, VBT predicts the K-Cs salt to be the most stable composition, where the salt-inclusion consists of Cs_6_K_2_Cl_2_ in the monoclinic form and KK_1.8_Cs_4.2_F in the orthorhombic form. DFT also predicts the monoclinic Cs_6_K_2_Cl_2_ salt-inclusion germanate structure to be the most stable. VBT suggests that the increase in silver content leads to less stable structures, as the formation energetics of silver ions is much larger than that of any of the alkali metals. For the orthorhombic structures calculated using both VBT and DFT (which include the following salt structures: KK_6_Cl, KK_6_Br_0.5_F_0.5_ and Na_0.9_Rb_6.1_F), the Na_0.9_Rb_6.1_F structure was found to be most stable by both VBT and DFT, where DFT treated the salt-inclusion as fully occupied Na_1_Rb_6_F. The remaining two structures are comparable, differing only in the variation of the halide (KK_6_Cl vs KK_6_Br_0.5_F_0.5_) with the mixed Br-F halide calculated to be more stable by DFT, which is the reverse for the VBT results, although both methods each predict very similar energies. Note that the DFT calculations for partial/mixed occupancies can be problematic as they demand significantly larger unit cells which are prohibitively computationally expensive, since both structure types include salts that have partial occupancies, only half of both the monoclinic and orthorhombic SIMs could be treated with DFT. The hexagonal structure with lower germanate content is predicted to have the least negative formation enthalpy of the germanate compounds, indicating that the uranium rich composition is significantly less stable than the other synthesized framework compositions. This is analogous to the uranyl silicate results, where Si-rich (or U-poor) frameworks are more stable than the uranium rich compositions for frameworks of identical charge.

With respect to the rare earth SIMs, experimental information regarding the formation enthalpies of one of the Ln-silicate structures, [K_2_K_7_F_2_][Gd_3_Si_12_O_32_], is reported^26^. The VBT Δ_*f*_*H*°_298.15_ value from the elements for the SIM is in good agreement with that obtained by drop solution calorimetry (Table 4). Both VBT and DFT predict that the Eu-containing silicate is more stable than its Gd-analogue, however DFT under-predicts the values compared to experiment (for Gd-SIM) and VBT. For all of the SIMs considered here, VBT generally predicts more negative enthalpies of formation compared to DFT, however general trends are in agreement for the silicates, germanates and Ln-silicates.

Note that the formation enthalpies were calculated using V_m_, from DFT relaxed structures, if experimental data on the crystal structure is lacking. A comparison in the the results from using V_m_, values from experimental and DFT computed structures for the uranyl silicate and germanate systems is found in Fig. 4. Overall, the volumes calculated with DFT lead to minor differences in the VBT computed energies, with a variation of no more than 2 percent. Most of the values computed with DFT-determined volumes are more negative (more stable) than those computed with experimental values for both silicates and germanate structures.

The formation enthalpies for DFT are calculated in vacuum at 0 K, however to include temperature dependence and entropic contributions are out of the scope of this work as they are too computationally demanding and not every salt-inclusion can be treated since partial occupancies pose a problem when generating the structures. VBT does however, produce entropic values that allow calculating the Gibbs energy of formation of each of the respective compositions (Table 4 and Fig. 5). The trends for the Gibbs energies remain consistent with those calculated for the formation enthalpies in that the silicates are found to be the more thermodynamically stable structures, except for one composition, which has a much smaller ***V***_***m***_ and salt-inclusion compared to the rest of the structures considered. Similarly, more negatively charged framework structures have increased stability, where the impact of the overall charge of salt-inclusion influences this stability, i.e., more ions within the salt-inclusion increase the ionic strength factor, which contributes to the lattice potential used for these calculations.

## Conclusions

In this work we compute relative stabilities of complex hierarchical structures for waste sequestration using computationally inexpensive techniques that rely on sound thermodynamic correlations. The enthalpies and Gibbs energies of formation of 24 SIMs were calculated using VBT methods and compared to the enthalpies of formation from DFT and experimental results when available. VBT and DFT results were in closest agreement for the smaller framework silicate structure, whereas DFT in general predicts less negative enthalpies across all SIMs, regardless of framework type. The uranyl-germanate structures were found to be slightly less thermodynamically stable than their silicate analogues. Both methods predict the Ln-silicates to be the most stable of the comparable structures calculated, with VBT results being in good agreement with an available experimental value from drop solution calorimetry. Additionally, DFT was used to calculate some of the framework components used in the thermochemical cycles for the volume-based methods. This allowed for a more physical representation of the structural units seen in experiment. As auxiliary information on SiO/SiO_2_ and GeO/GeO_2_ building blocks are limited to singly charged species, DFT aids in obtaining information on higher oxidation states, which are necessary to charge balance these complex systems. While certain thermochemical cycles yield VBT values in better agreement with DFT results, discrepancies still exist between the absolute values of both methods. Similarly, implementation of U_eff_ in DFT, as is standard for f-electron systems, leads to lower (more negative) formation energies, however this does not resolve the disparity as the values calculated with *U*_eff_ = 4.0 eV are only about ~100 kJ/mol lower than those computed using *U*_eff_ = 0 eV. Improvements in the thermochemical cycles of VBT and manipulation of the U_eff_ values might produce better agreement.
